# A national study exploring the association between triglyceride-glucose index and risk of hyperuricemia events in adults with hypertension

**DOI:** 10.1016/j.pmedr.2024.102763

**Published:** 2024-05-17

**Authors:** Leixia Wang, Jianqian Chao, Na Zhang, Yanqian Wu, Min Bao, Chenyuan Yan, Tong Chen, Xinyue Li, Yiqin Chen

**Affiliations:** a Health Management Research Center, School of Public Health, Southeast University, Nanjing, China; b Shenzhen Institute of Advanced Technology Chinese Academy of Sciences, Shenzhen, China; c School of Clinical Medicine, Southwest Medical University, Luzhou, China; d School of Public Health, Southwest Medical University, Luzhou, China

**Keywords:** Hyperuricemia, Triglyceride-glucose index, Hypertension, Insulin resistance, NHANES

## Abstract

**Background:**

The triglyceride-glucose (TyG) index has been recommended as a practical surrogate of insulin resistance (IR). However, the association between the TyG index and hyperuricemia among adults with hypertension remains to be elucidated.

**Methods:**

We included and analyzed 3134 HTN patients and 4233 non-HTN participants from the cross-sectional 2013–2018 U.S. National Health and Nutrition Examination Surveys (NHANES). Multivariable logistic regression and restricted cubic splines (RCS) were used to explore the association between the TyG index and hyperuricemia. Stratifed analyses were performed to assess the association in populations with different subgroups of hypertension.

**Results:**

The prevalence of hyperuricemia was higher in HTN patients (28.00 %) than in non-HTN participants (12.47 %). The multivariable logistic regression showed that the TyG index was significantly associated with hyperuricemia. After multivariable adjustment, higher TyG index levels were found to be associated with a higher prevalence of hyperuricemia in HTN patients (OR: 2.39, 95 % CI: 1.37–4.17, *P*_trend_ < 0.001) and non-HTN participants (OR: 2.61, 95 % CI: 1.45–4.69, *P*_trend_ < 0.001). Restricted cubic spline regression showed linearity of the associations between the TyG index and hyperuricemia (*p*-nonlinear > 0.05). In the subgroup analysis suggested that the positive association seemed to be strong among male, alcohol use, and diabetes group (*P* for interaction < 0.05).

**Conclusions:**

TyG index, a practical surrogate of IR, was linearly and positively associated with hyperuricemia in HTN and non-HTN participants. Proactive measures are needed to prevent the comorbidity of IR-driven hyperuricemia in the future.

## 1 Introduction

Hyperuricemia (HUA) is a common form of chronic metabolic disorder caused by an imbalance between uric acid production and excretion (Li et al., 2020, Copur et al., 2022). An epidemiological survey showed that approximately 21 % of U.S. adults had hyperuricemia in 2016, and the prevalence is generally on the rise (Chen-Xu et al., 2019). While the vast majority of patients with hyperuricemia are asymptomatic, prolonged periods of elevated uric acid levels can lead to the deposition of monosodium crystals of urate, which can damage joint structures and eventually lead to gout (Dehlin et al., 2020, Major et al., 2018, Dalbeth et al., 2021). Furthermore, hyperuricemia is also thought to be associated with an increased risk of chronic conditions, including renal diseases, cardiovascular diseases (CVD), and metabolic syndrome (Yu and Cheng, 2020, Borghi et al., 2022). Therefore, it is quite important to identify the high-risk population of hyperuricemia and provide early prevention.

Insulin resistance (IR) is a pathological condition of decreased sensitivity and responsiveness to the action of insulin caused by a variety of factors including genetics, environment, illness, and diet (Lee et al., 2022, Yang et al., 2018). Numerous studies have confirmed that insulin resistance plays an important role in the development and prognosis of cardiovascular diseases (Haffner, 2003, Louie et al., 2023). The triglyceride-glucose (TyG) index is derived from the logarithmic transformation of the product of fasting blood glucose and triglycerides. In clinical practice, the TyG index has been identified as an inexpensive and reliable surrogate of IR compared with the traditional homeostasis model (HOMA-IR) (Vasques et al., 2011).

Several epidemiological studies have investigated the association between the TyG index and hyperuricemia in different populations. A retrospective case-control study found that the TyG index was an independent risk factor for hyperuricemia in patients with non-alcoholic fatty liver disease (NAFLD) (OR[95 %CI]:2.00[1.38,2.91]) (Qi et al., 2023). In addition, in a longitudinal study, the TyG index was also positively correlated in diabetic kidney disease (DKD) adults [15]. Recent studies have revealed the correlation between the TyG index and hyperuricemia in general populations, and the TyG index was more closely associated with hyperuricemia than obesity indices (Zhu et al., 2020, Han et al., 2023).

Although several studies have explored the correlation between the TyG index and hyperuricemia risk, it is unclear whether this risk also exists in hypertensive populations, who tend to have longer disease duration and more comorbidities. From all those facts, we hypothesized that the TyG index of hypertensive adults would positively correlate with the risk of hyperuricemia. Therefore, we conducted this study to investigate the prevalence of hyperuricemia in a nationally representative sample of U.S. adults and to explore whether there is a positive correlation between the TyG index and risk of hyperuricemia in hypertension (HTN) and non-hypertension (non-HTN), in the hope of providing some information on the prevention of hyperuricemia.

## 2 Methods

### 2.1 Study population and data definition

The National Health and Nutrition Examination Survey (NHANES) is an ongoing cross-sectional study, which employed a complex, multistage probability design to collect nationally representative nutrition and health-related data on the civilian non-institutionalized U.S. population. The survey combined personal structured interviews, mobile physiological measurements, as well as laboratory tests. All participants provided written informed consent, and the study protocols were approved by the National Center for Health Statistics (NCHS) Research Ethics Review Board (http://www.cdc.gov/nchs/nhanes/irba98.htm).

**Fig. 1:**
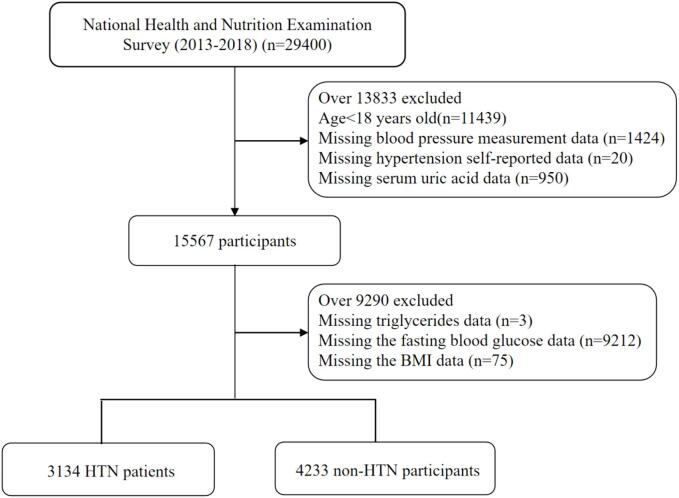
Flowchart of study population selection, NHANES 2013–2018.

In the current study, we used the data from three consecutive cycles of NHANES (2013–2014, 2015–2016, and 2017–2018), which provided information on serum uric acid value. HTN was defined as self-reported doctor diagnosis of hypertension, use of antihypertensive medications, or systolic blood pressure (SBP) ≥ 140 mm Hg or diastolic blood pressure (DBP) ≥ 90 mm Hg referring to the 2017 American Heart Association (AHA) recommendations (Cuspidi et al., 2018). Overall, there were 29,400 participants in NHANES from 2013 to 2018. We selected 7387 participants aged 18 years and older who had completed measurements of serum uric acid, triglyceride, fasting blood glucose, BP and BMI. We also excluded 20 participants without self-reported diagnosis HTN information. Finally, we included a total of 7367 participants, of whom 3134 were HTN patients and 4233 were non-HTN (Fig. 1).

### 2.2 Ascertainment of the TyG index

Blood specimens were collected at mobile examination centers (MECs) by NCHS-trained professionals and stored under appropriate frozen conditions until shipped to laboratory for further testing. Glucose concentrations were analyzed as part of the routine serum biochemistry profile using the oxygen rate method employing a Beckman Oxygen electrode. Triglyceride was measured on the Roche Cobas 6000 chemistry analyzers. Details of the specimen measurement and quality control methods are provided in the NHANES Laboratory/Medical Technician Procedures Manual (LPM), which is available on the website. The TyG index was obtained by using the equation ln (fasting triglyceride [mg/dL] × fasting blood glucose (FBG) [mg/dL]/2).

### 2.3 Hyperuricemia assessment

Hyperuricaemia was determined by serum uric acid (SUA) values obtained from laboratory data. Well-trained professionals used the Beckman Coulter UniCel® DxC800 with a timed endpoint method to measure the concentration of SUA. In this study, the diagnostic criteria for hyperuricemia were serum uric acid levels ≥ 7 mg/dL (420 µmol/L) in males and ≥ 6 mg/dL (360 µmol/L) in females (Bardin et al., 2014).

### 2.4 Covariates

Sociodemographic characteristics were obtained from standardized questionnaires in NHANES 2013–2018, including age, gender (males, females), marital status (married, divorce), education (high school below, completed high school and more than high school), race/ethnicity (Mexican American, non-Hispanic white, non-Hispanic black and other races). Information on smoking status, alcohol use and physical activity were obtained through participant self-report. Smoking status was classified as never smokers (smoked < 100 during lifetime), former smokers (smoked > 100 cigarettes but had quit), or current smokers (smoked > 100 cigarettes without quitting). Alcohol use was derived from two 24-hour dietary recall data, and participants were considered drinkers if they reported alcohol consumption on at least one 24-hour dietary recall. Physical activity was assessed by the Global Physical Activity Questionnaire (GPAQ) in NHANES. According to metabolism equivalent (MET) intensity, we categorized physical activity as inactive group (<3 METs), moderate active group (3–6 METs), and vigorous active group (>6 METs). Body mass index (BMI), waist circumference (WC), total cholesterol (TC), triglycerides (TG), high-density lipoprotein cholesterol (HDL-C), low-density lipoprotein cholesterol (LDL-C), and Haemoglobin A1c (HbA1c) were obtained from laboratory test data. participant's self-reported comorbidities, such as diabetes, renal failure, kidney stones, heart failure and stroke, were obtained from questionnaire data. In addition, HbA1c ≥ 6.5 % (48 mmol/mol) or FBG ≥ 126 mg/dL could also be considered as a participant having diabetes.

### 2.5 Statistical analysis

Given the complex survey design considerations of NHANES, we applied appropriate sampling weights for our analysis. Continuous variables were expressed as means with standard deviations (SDs) or medians with interquartile ranges (IQRs), and categorical variables were expressed as numbers (percentages). The difference between the HTN and the non-HTN group was compared by Student’s *t*-test (continuous variables with normal distribution) or Mann–Whitney *U* test (continuous variables with non-normal distribution), and χ^2^ test (categorical variables).

We applied multivariable logistic regression analysis to evaluate the association between the TyG index and hyperuricemia in HTN and non-HTN participants, the results of the model were reported through odds ratios (ORs) and 95 % confidence intervals (CIs). We fitted three statistical models. Model 1 was unadjusted. Model 2 was adjusted for sex (male or female), age (continuous), education (less than high school, completed high school, or more than high school), race/ethnicity (Mexican American, non-Hispanic white, non-Hispanic black, or other), current smoking (yes or no), alcohol use (yes or no), and physical activity (inactive group, moderate group, or vigorous group). Model 3 was further adjusted for BMI, WC, FBG, TC, TG, HDL-C, LDL-C, HbA1c, and self-reported comorbidities (DM, renal failure, kidney stones, heart failure, stroke, hepatopathy, and hyperlipidemia). Quartiles of TyG index levels were determined based on the distribution in the study, and the linear trend was calculated by assigning a median value to each category as a continuous variable. Additionally, we performed subgroup analyses based on age, sex, education, race/ethnicity, smoking status, alcohol use, diabetes, and BMI in HTN patients. To estimate the dose–response relationship between the TyG index and hyperuricemia in the study population, we employed a restricted cubic spline regression model with three knots at the 5th, 50th, and 95th percentiles. Data processing and analysis were performed using R version 4.3.0, along with Storm Statistical Platform (https://www.medsta.cn/software). A two-tailed value of *P* < 0.05 was considered statistically significant.

## 3 Results

### 3.1 Participants characteristics

General characteristics and laboratory parameters of the 7367 participants, 3134 with and 4233 without hypertension, are presented in Table 1. The prevalence of hyperuricemia in hypertensive patients was 28.00 %, which is significantly higher than that in the population without hypertension (12.00 %). Among 3,134 patients with hypertension (mean age, 57.51 years; 50.02 % males), the median (interquartile range) TyG index was 8.79 (8.39, 9.26). Compared with non-hypertensive participants, those with hypertension were more likely to be non-Hispanic white, older, have lower leisure-time physical activity, higher BMI and education level, and have a higher prevalence of DM, renal failure, kidney stones, heart failure, stroke, hepatopathy, and hyperlipidemia (*P* < 0.05). In addition, there were significant differences in WC, FBG, TC, TG, HDL-C, LDL-C, HbA1c, and SUA values between the two groups (*P* < 0.05).

**Table 1:** Comparison of baseline characteristics between adults with and without hypertension in the U.S. from NHANES 2013–2018.

Participant characteristics	HTN (n = 3134)	Non-HTN (n = 4233)	*P*-value
HUA (n, %)	879 (28.00)	528 (12.00)	<0.001
Age (years, mean ± SD)	57.51 ± 0.37	47.58 ± 0.40	<0.001
Males, (n, %)	1,542 (50.02)	2,019 (49.01)	0.209
Married, (n, %)	1,833 (64.06)	2,507 (63.01)	0.543
Education			<0.001
<High school	338 (5.62)	300 (3.90)	
Completed high school	428 (10.02)	615 (10.01)	
More than high school	2366 (84.36)	3317 (86.09)	
Race/ethnicity, (n, %)			<0.001
Mexican American	368 (6.00)	736 (11.00)	
Non-Hispanic white	1218 (68.00)	1544 (64.04)	
Non-Hispanic black	825 (13.00)	705 (8.33)	
Other	723 (13.00)	1248 (16.63)	
Current smoking, (n, %)	610 (19.01)	795 (18.04)	0.374
Alcohol use, (n, %)	281 (11.02)	344 (11.01)	0.216
Physical activity level, (n, %)			<0.034
Inactive	1885 (53.90)	2351 (50.99)	
Moderate	656 (24.88)	913 (24.04)	
Vigorous	593 (21.22)	969 (24.97)	
BMI (kg/m^2^) [M, (P25, P75)]	30.00 (26.00, 35.00)	27.00 (23.00, 31.00)	<0.001
WC (cm, mean ± SD)	106.10 ± 0.45	99.98 ± 0.39	<0.001
FBG (mg/dL)	116.96 ± 0.97	108.24 ± 0.48	<0.001
TC (mg/dL, mean ± SD)	190.74 ± 1.16	188.69 ± 0.89	<0.001
TG (mg/dL, mean ± SD)	145.39 ± 2.71	128.07 ± 1.84	<0.001
HDL-C (mg/dL, mean ± SD)	53.752 ± 0.59	54.87 ± 0.38	0.053
LDL-C (mg/dL, mean ± SD)	107.18 ± 0.93)	107.02 ± 0.72	0.564
HbA1c (%)	5.95 ± 0.03	5.65 ± 0.02	<0.001
SUA (mg/dL) [M, (P25, P75)]	5.70 (4.70, 6.80)	5.19 (4.30, 6.00)	<0.001
TyG index [M, (P25, P75)]	8.79 (8.39, 9.26)	8.44 (8.07, 8.88)	<0.001
Self-reported comorbidities, (n, %)			
DM	1127 (31.00)	580 (11.01)	<0.001
Renal failure	214 (5.21)	50 (1.12)	<0.001
Kidney stones	400 (14.00)	320 (8.22)	<0.001
Heart failure	220 (5.41)	34 (0.61)	<0.001
Stroke	223 (5.92)	60 (1.11)	<0.001
Hepatopathy	208 (6.91)	127 (2.61)	<0.001
Hyperlipidemia	2637 (86.01)	2558 (71.01)	<0.024

All estimates accounted for complex survey designs. Data are described as mean ± standard deviation (SD) or medians (interquartile ranges) for continuous variables, or as numbers (weighted percentages) for categorical variables.

HTN, hypertension; HUA, hyperuricaemia; TyG index, triglyceride-glucose index; BMI, body mass index; WC, waist circumference; TC, total cholesterol; TG, triglycerides; HDL-C, high-density lipoproteins cholesterol; LDL-C, low-density lipoprotein cholesterol; HbA1c, Haemoglobin A1c; FBG, fasting blood glucose; SUA, serum uric acid; DM, diabetes mellitus.

### 3.2 Relationship of TyG index with hyperuricemia events

**Fig. 2:**
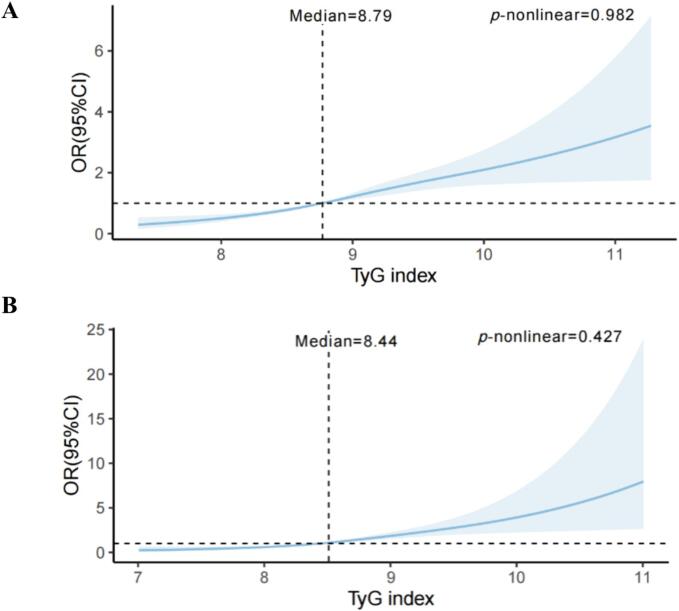
Dose-response association between TyG index and hyperuricemia in U.S. adults (A) with and (B) without hypertension, respectively. Adjusted for sex, age, education, race/ethnicity, smoking, alcohol use, physical activity, BMI, WC, FBG, TC, TG, HDL-C, LDL-C, HbA1c, and self-reported comorbidities (DM, renal failure, kidney stones, heart failure, stroke, hepatopathy, and hyperlipidemia). The blue line shows the OR value and the blue shaded areas indicate the 95 % CI. OR, odds ratio; CI, confidence interval; TyG index, triglyceride-glucose index; *P* nonlinear > 0.05 indicates a significant linear relationship. (For interpretation of the references to colour in this figure legend, the reader is referred to the web version of this article.)

Table 2 shows the OR value and correlation trends of the TyG index with hyperuricemia events after the modeling logistic regression. 879 and 528 hyperuricemias were identified in HTN and non-HTN subjects, respectively. Among participants with hypertension, higher TyG index was significantly associated with elevated risk of hyperuricemia. After adjusting for all covariates, the positive association remains statistical significance.. The multivariable-adjusted ORs (95 % CIs) across quartiles of the TyG index were 1.00 (reference), 1.44 (0.99, 2.09), 1.71 (1.14, 2.58), and 2.39 (1.37,4.17) (*P* trend < 0.001). Similarly, hyperuricemia was associated with the higher quartiles of the TyG index among participants without hypertension. After multivariable adjustment, we found a significantly higher prevalence of hyperuricemia in non-HTN participants with the highest quartile of TyG index than in those with the lowest (OR: 2.61, 95 %CI: 1.45–4.69, *P* trend < 0.001). We also found an interaction effect between hypertension (with or without) and TyG index on hyperuricaemia (*P* for interaction < 0.05). The results of dose–response relationship analysis with restricted cubic spline (RCS) were plotted in Fig. 2. Higher TyG index levels were linearly positively associated with the prevalence of hyperuricemia both in HTN and non-HTN individuals (*P* for nonlinear > 0.05).

**Table 2:** The association between TyG index and hyperuricemia among HTN and non-HTN participants, NHANES 2013–2018.

**TyG index**	**HUA events/total**	**Model 1**	**Model 2**	**Model 3**
		**OR (95CI)**	**OR (95CI)**	**OR (95CI)**
HTN patients				
Quartile 1 (<8.39)	159/833	1.00	1.00	1.00
Quartile 2 (8.39–8.79)	205/773	1.61 (1.14, 2.28)	1.62 (1.11, 2.34)	1.44 (0.99, 2.09)
Quartile 3 (8.80–9.26)	247/788	2.07 (1.50, 2.84)	2.15 (1.51, 3.04)	1.71 (1.14, 2.58)
Quartile 4 (>9.26)	268/740	2.68 (1.87, 3.84)	2.86 (1.92, 4.27)	2.39 (1.37, 4.17)
*P* trend		<0.001	<0.001	<0.001
Non-HTN participants				
Quartile 1 (<8.17)	80/1374	1.00	1.00	1.00
Quartile 2 (8.17–8.59)	107/1110	1.52 (1.01, 2.29)	1.63 (1.07, 2.47)	1.19 (0.76, 1.86)
Quartile 3 (8.60–9.04)	157/938	2.68 (1.79, 4.01)	2.96 (1.96, 4.47)	1.77 (1.11, 2.83)
Quartile 4 (>9.04)	184/811	4.01 (2.75, 5.85)	4.56 (3.00, 6.94)	2.61 (1.45, 4.69)
*P* trend		<0.001	<0.001	<0.001

Data are presented as OR (95 % CI) unless otherwise noted. Model 1: Unadjusted; Model 2:Adjusted for sex, age, education, race/ethnicity, smoking, alcohol use, and physical activity. Model 3:Model 2 + BMI, WC, FBG, TC, TG, HDL-C, LDL-C, HbA1c, and self-reported comorbidities (DM, renal failure, kidney stones, heart failure, stroke, hepatopathy, and hyperlipidemia).

### 3.3 Subgroup analysis among hypertensive patients

**Fig. 3:**
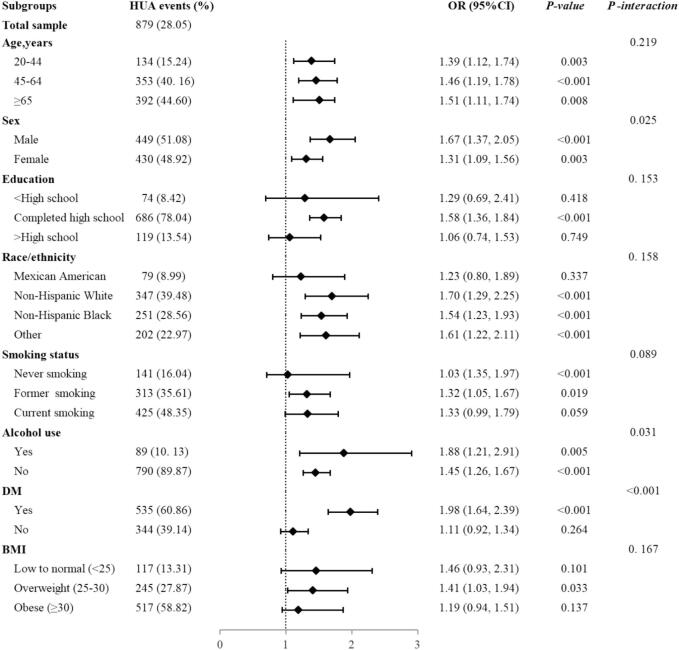
Subgroup analysis of TyG index and hyperuricemia in U.S. adults with hypertension, NHANES 2013–2018. DM, diabetes mellitus; BMI, body mass index; TyG index, triglyceride-glucose index; OR, odds ratio; CI, confidence interval. All ORs were adjusted for sex, age, education, race/ethnicity, smoking, alcohol use, physical activity, BMI, WC, FBG, TC, TG, HDL-C, LDL-C, HbA1c, and self-reported comorbidities (DM, renal failure, kidney stones, heart failure, stroke, hepatopathy, and hyperlipidemia).

We performed stratified analyses to further reveal the relationship between the TyG index and hyperuricaemia in different subgroups among HTN patients (Fig. 3). Significant associations of TyG index with hyperuricemia risk were found in each stratum of age, sex, and alcohol use (*P* < 0.05). In addition, we found significant interactions in three subgroups: sex (male vs. females), alcohol use (yes vs. no), and DM (yes vs. no). Male with hypertension had a great risk (OR = 1.67, 95 %CI:1.37–2.05) of developing hyperuricaemia than female (OR = 1.31, 95 %CI:1.09–1.56, *P* for interaction = 0.025). Hypertensive patients who drink alcohol are at greater risk (OR = 1.88, 95 %CI:1.21–2.91) of developing hyperuricaemia compared to non-drinkers (OR = 1.45, 95 %CI:1.26–1.67, *P* for interaction = 0.031). Similarly, the association was strong comfirmed in the diabetes group compared to the non-diabetes group (OR = 1.98, 95 %CI:1.64–2.39, OR = 1.11, 95 %CI:0.92–1.34, *P* for interaction < 0.001).

## 4 Discussion

In this nationally representative study, the relationship between the TyG index and hyperuricemia was evaluated. The main finding of this study is that the TyG index was positively associated with hyperuricemia in the American adult population. The restricted cubic spline indicated that the association was linear across the TyG range. This finding was also validated in adjusting for other covariates. Moreover, the subgroup analysis suggested that the positive association seemed to be strong among male, alcohol use, and diabetes group (*P* for interaction < 0.05). Previous studies have examined the relationship between TyG index and hyperuricaemia among patients with hypertension in clinical setting (Liu et al., 2023, Dong et al., 2022 Jan 2). To our knowledge, this is the first study to examine the relationship of the TyG index with hyperuricemia in U.S. adults with HTN using the NHANES database, which is derived from a national, community-oriented survey.

Hyperuricemia plays a pathogenic role in the progression of CVD. A prospective cohort study showed that hyperuricemia increased the risk of HTN 1.17-fold (Sundström et al., 2005). A 9-year follow-up study also indicated a positive correlation between serum uric acid level and HTN (Mellen et al., 2006). The presumed mechanism by which hyperuricemia contributes to CVD involves renin-angiotensin system activation, IR, inflammatory reaction, and oxidative stress (Waheed et al., 2021). We found that 28.00 % of HTN patients had hyperuricemia, which supports the evidence that hypertensive patients are at high risk and need early identification and control. Furthermore, in the setting of IR and HTN, many patients with hyperuricemia may be at risk for being “rapid progressors” to more severe comorbidities (Johnson et al., 2018).

Insulin is a peptide hormone consisting of 51 amino acids that is important for energy metabolism and maintaining homeostasis. IR, originally used to describe diabetics who require high doses of insulin (Tahapary et al., 2022). There is increasing evidence that IR is also associated with metabolic syndrome, which includes hyperlipidemia, hypertension, and obesity, leading to an increased risk of cardiovascular disease. A bidirectional Mendelian randomization study assessing the causal relationship between IR and hyperuricemia and gout based on genome-wide association data and the UK Biobank database suggested that hyperinsulinemia leads to hyperuricemia and that reduction of insulin resistance may reduce serum urate concentrations and the risk of gout (McCormick et al., 2021).

Currently, the gold standard method for IR is the hyperinsulinemic euglycemic clamp (HEC) method, but this method is invasive and labor intensive (Tahapary et al., 2022, Muniyappa et al., 2008). As a simple surrogate for IR, multiple studies have validated the effectiveness of the TyG index in the assessment of IR and prevention of its IR-driven comorbidities. However, few studies have focused on the relationship between the TyG index, a practical surrogate of IR, and hyperuricemia. In a population-based nationwide cohort study of 5269 Chinese individuals (aged ≥ 45 years), Han Y et al found that those with low-to-high TyG index had a significantly higher risk of hyperuricemia compared to individuals with maintained low levels of TyG index, which was consistent with the results of this study (Han et al., 2023). Another study in the medical examinations population also found that the TyG index was more closely related to hyperuricemia than obesity indices (e.g., body roundness index, visceral adiposity index, and waist-to-height ratio) (Kahaer et al., 2022). The positive association between IR and hyperuricemia has been attributed to several mechanisms. Firstly, IR destroys oxidized phosphates, leading to an increase in the level of adenosine, which is sodium-retentive and therefore reduces the excretion of uric acid. In addition, IR enhances the inflammatory cascade and leads to hepatic dysfunction, which increases xanthine oxidoreductase activity, resulting in hyperuricaemia (Han et al., 2023, Waheed et al., 2021, Yanai et al., 2021, McCracken et al., 2018). Our subgroup analysis showed that hypertensive patients who were male, alcoholics or had comorbid DM had a higher risk of hyperuricemia.

Hypertension is a global public health concern. Patients with hypertension are often complicated by a variety of metabolic disorders, including hyperlipidemia, obesity and DM. We found that 86 % of the hypertensive patients were comorbid with hyperlipidemia and nearly one-third had DM. It is acknowledged that TyG index is positively associated with the prevalence of DM and hypertension (Luo et al., 2022). The result of this study suggested that the TyG index was linearly and positively associated with the risk of hyperuricaemia in patients with HTN. Furthermore, subgroup analysis was performed to validate the universality of the association. Our study had several strengths. Based on a cross-sectional design, the current study is among the first to evaluate the associations of TyG index with hyperuricemia in a nationally representative sample of U.S. adults with HTN. Moreover, multiple potential confounders were carefully adjusted, including sociodemographic characteristics, lifestyle factors, and various physiological and biochemical parameters. We investigated the relationship between the TyG index and hyperuricemia in hypertensive patients, a specific group. This demonstrates that the TyG index may be useful in identifying HTN patients at high risk for hyperuricemia and guiding further detection and treatment. Despite the strengths, several limitations should also be noted. First, the NHANES is an observational study design, causality inference between TyG index and hyperuricemia could not be established. Thus, further prospective cohort studies are needed. Second, although several important covariates were adjusted, we cannot completely eliminate residual effects of other potential confounders, such as purine-rich diet and medication use and family history of hyperuricemia.

## 5 Conclusions

In a nationally representative sample of U.S. adults with HTN, we found the TyG index, a practical surrogate of IR, was linearly and positively associated with the risk of hyperuricemia. Our study provides some evidence for the value of the TyG index in the prevention of hyperuricemia in hypertensive populations. Proactive measures are needed to prevent the comorbidity of IR-driven hyperuricemia in the future.

## Funding

This work was supported by the Key Projects of the National Social Science Foundation of China (No. 22AZD082).

## Ethical approval and consent to participate

The study protocol for the US NHANES was approved by the US NHANES institutional review board and National Center for Health Statistics Research ethics review board. All participants provided written informed consent. Institutional review board approval was waived for this analysis because of the publicly available and deidentified data.

## CRediT authorship contribution statement

**Leixia Wang:** Writing – review & editing, Writing – original draft, Investigation, Conceptualization. **Jianqian Chao:** Supervision, Resources, Project administration, Funding acquisition. **Na Zhang:** Software, Methodology, Investigation. **Yanqian Wu:** Validation, Software. **Min Bao:** Visualization, Validation, Software. **Chenyuan Yan:** Software, Project administration. **Tong Chen:** Methodology, Formal analysis. **Xinyue Li:** Formal analysis, Data curation. **Yiqin Chen:** Visualization, Software.

## Declaration of competing interest

The authors declare that they have no known competing financial interests or personal relationships that could have appeared to influence the work reported in this paper.

## Data Availability

Data will be made available on request.
